# Supplementary material for: Gut Microbiome and Associated Metabolites Following Bariatric Surgery and Comparison to Healthy Controls
Source: Microorganisms. 2023 Apr 26;11(5):1126. doi: 10.3390/microorganisms11051126 (PMC10223326; doi:10.3390/microorganisms11051126)
Supplement: Supplementary file 1 [file microorganisms-11-01126-s001.zip › microorganisms-2281746-supplementary.pdf]

Table S1. Characteristics of healthy cohort at baseline and 6-month follow-up.

	Healthy		
	**Baseline**	**Follow-up**	***p* Value**
N	59	53	
Age, years	56.0 ± 9.2	57.1 ± 8.9	0.52
BMI, kg/m^2	26.4 ± 3.9	26.5 ± 3.8	0.89
Men, n (%)	11 (18.6)	11 (20.8)	1.0
**Smoker**			
Smoker, n, %			
Current	0 (0)	0 (0)	-
Previous	21 (35.6)	18 (34.0)	-
Never	38 (64.4)	35 (66.0)	-

Values are mean ± SD, n, n (%).

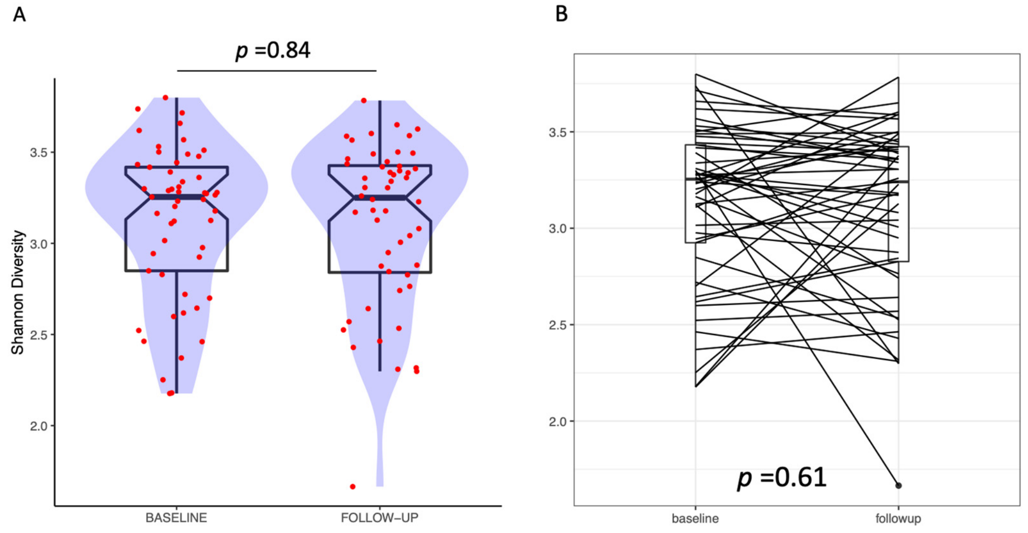

Figure S1. Analysis of microbiome diversity and composition in healthy volunteers at baseline and 6-months follow-up. (**A**) Barplots overlaid with density plots showing Shannon diversity at baseline and follow-up for all healthy volunteers. Red dots represent the diversity value for each sample analysed. (**B**) Barplots showing the alpha diversity (Shannon Index) for the paired data obtained at baseline (left) and follow up (right). Samples collected from the same patient are connected with a solid line.
